# Book Reviews

**Published:** 1905-01

**Authors:** 


### BOOK REVIEWS.

A Textbook of Pathology. By Joseph McFarland, M.D., Professor of Pathology and Bacteriology in the Medico-Chirurgical College, Philadelphia. Octavo volume, 818 pages, with 350 illustrations. Philadelphia, New York, London: W. B. Saunders & Co. 1904. (Cloth, $5.00 net; sheep or half morocco, $6.00 net.)

A new work on pathology coming at a time when the field is already pretty well occupied, is likely to stimulate inquiry as to its necessity no less than as to its quality. In order to establish the one, it becomes requisite to make good the other. It would be a wearisome if not unprofitable task for us to make a critical or analytical statement in detail of the quality of McFarland's labors in the field of pathology, but we shall content ourselves with pointing out here and there special qualities of his textbook.

As an army partakes of the qualification of its commander, so does a book become the expression of the man behind the pen. The great Napoleon said, "In war men are nothing; a man is everything!" So, too, in medical literature books are nothing, unless the authors are capable of commanding the respect and attention of the studying and thinking portion of the medical profession. Happily, this author is such a man. Having taught pathology and bacteriology for thirteen years and being a diligent student of the theme delineated in this treatise he commands attention at once.

It would be well, in our view, if pathology were accorded third place in the preliminary studies of the neophyte. Human anatomy, by common consent and time-honored custom, comes first; then human physiology follows; and why, will some one explain, should not the anatomy of disease—pathology—become the third topic in the order of sequence and of importance? It appears logical that such a plan should be adopted in the school curriculum, and next after pathology physiological chemistry might follow with propriety. This would endow pathology with its proper importance early in the mind of the pupil; he would learn early in his career to cultivate one of the most important topics in medicine and make it an agreeable instead of a hated study.

McFarland addresses his textbook to students of medicine in particular, hence has laid down the principals of pathology in impressive fashion, paying attention to established facts rather than to unsettled or abstruse questions. The work is partitioned into two grand divisions: (1) general, and (2) special pathology. The first section deals with disease processes common to the whole organism, such as the causes of disease, defects of development, pathology of nutrition, characteristics of cell life, progressive tissue changes, immunity, infection, and the like; while the second subdivision treats of diseased conditions in the special organs, beginning with the blood and the cardiovascular system, going on through the digestive, respiratory, nervous, and genito-urinary systems; also, intercurrently considering diseases of the skin, the glandular structures, and the motor apparatus.

The comprehensiveness of the book will become apparent from the foregoing epitome of its contents. To obviate voluminousness two kinds of type are used,—the larger for more important material, the smaller for less weighty portions of the text. Illustrations have been used with considerable freedom and discretion, some of the pictures being original, while others are borrowed from authoritative sources, all of which deserve approbation.

It would appear, after a reasonably careful examination of this treatise, that it is not overstating its merits to predict for it the approval of teachers of pathology, and that they will adopt it as a textbook in their undergraduate courses.

---

The Theory and Practice of Infant Feeding. With Notes on Development. By Henry Dwight Chapin, A.M., M.D., Professor of Diseases of Children at the New York Post-Graduate Medical School. Second edition, revised. Octavo, pp. 353. Illustrated. New York: William Wood & Company. 1904. (Cloth, $2.25.)

It is quite evident from an examination of this treatise that the last word has not been said concerning the feeding of infants.

The underlying principle in Chapin's investigations is that food must be adapted to the particular species according to the digestive apparatus of the animal, and that the purely chemical basis is untenable. No doubt there always will be a contention between the advocates of the standards set up between breast milk and cow's milk, but recent discoveries pertaining to the latter, will serve to increase its favor for artificially fed infants.

The revisions in this edition are considerable, some portions of it having been rewritten, so that it may be said to represent the latest thought relating to infant feeding. The illustrations in such a work necessarily are limited in extent, but those chosen by Chapin serve the excellent purpose of object lessons as pertaining to the preparation of cow's milk for purposes of artificial feeding; while, again, the reproduced photographs of infants in the various stages of development are both entertaining and instructive. Without doubt, no treatise superior to Chapin's on infant feeding has been published.

---

A System of Practical Surgery. By Prof. E. von Bergmann, M.D., Berlin, Prof. P. von Bruns, M.D., Tübingen, and Prof. J. von Mikulicz, M.D., Breslau. Volume V. Surgery of the Pelvis and Genitourinary Organs. Translated and edited by William T. Bull, M.D., Professor of Surgery, College of Physicians and Surgeons, New York, and Edward Milton Foote, M.D., Instructor in Surgery. Royal octavo, 789 pages. Illustrated. New York and Philadelphia: Lea Brothers & Company. 1904. (Price: cloth, $6.00; leather, $7.00; half-morocco, $8.50 net.)

The fifth and final volume of this excellent system of surgery fully maintains the standard set by the earlier numbers in the von Bergmann series. It is devoted to the consideration of the surgery of the pelvis and genitourinary organs, excluding the sexual organs of woman.

The surgery of the kidney, which is attracting the special attention of surgeons, is delineated with minute detail in this book, as are also the several other important surgical problems of the pelvic cavity and genitourinary system. The first section, four chapters, presents malformations and diseases of the pelvis itself; then the pelvic contents are dealt with, the next in order of sequence being a section on malformations, injuries, and diseases of the anus and rectum. The surgical management of hemorrhoids and of cancer of the rectum will attract attention, as being the commoner diseases of this region which are presented to the surgeon for relief.

The third section includes abnormalities, injuries, and diseases of the kidney and ureter; the fourth presents abnormalities, injuries, and diseases of the prostate; the fifth, malformations, injuries, and diseases of the urethra; the sixth, malformations, injuries, and diseases of the penis; the seventh and final section deals with anomalies, injuries, and diseases of the scrotum, testicle, vas deferens, and seminal vesicle.

We have never seen the subjects which are considered in the latter three sections handled with so much minute care and attention to detail as in this treatise; especially is this the case with reference to the malformations, injuries and diseases of the penis.

Throughout the entire series of volumes comprised in this work there is evidence of great care in the expression of opinions regarding technical surgical procedures to be adopted in given conditions, only those of value according to the experience of the authors being advised. The illustrations, for the most part on wood, but frequently by colored plates and reproductions, are of a high order, and deserve notice as being of practical utility. Finally, the work is the exponent of all that is best in modern surgery from the German viewpoint,—than which there is none better.

---

NORMAL HISTOLOGY. By EDWARD K. DUNHAM, Ph.B., M.D., Professor of General Pathology, Bacteriology and Hygiene in the University and Bellevue Hospital Medical College, New York. New (third) edition, revised and enlarged. Octavo, 334 pages, with 260 illustrations. Philadelphia and New York: Lea Brothers & Company. 1904. (Cloth, $2.75 net.)

To instruct the undergraduate student adequately in histology, it is necessary that a good book of instructions should be placed in his hands at the beginning of his studies. Such a book Dunham has constructed, which is clearly proven by the favor with which it was received in the first instance, and the further token of its excellence by being summoned so early into its third edition.

The scheme of the author was considered novel when first announced, but it has stood the test of experience. In general the plan chosen was, first, the conception of the cell as the active constituent of the tissues; second, that the elementary tissues are composed of cells and intercellular substances, their differences depending upon the proportions of those constituents; third, that the structural details of the different tissues are intimately correlated to their usefulness, function and structures being mutually dependent but being two aspects of a single arrangement; and, fourth, that the ability of tissues to perform active functions is proportional mainly to the number or size of cells entering into their structure. This scheme is further elaborated by arbitrarily dividing the activities of the cells theoretically into four groups—namely, first, the reproductive, leading to the reproduction of new cells; second, the formative, through which the structural modifications resultant in different varieties of tissue are brought about; third, the nutritive, which maintain the integrity of already formed tissues; and, fourth, the functional activities, through which the tissues serve the whole organism of which they are constituent parts.

From the foregoing, it will be seen that the author is both original and scientific, and that he has put forth a book which every student of histology should take with him into the laboratory, which is the proper place in which to teach this topic. The illustrations contribute to the value of the book, which is not second to any yet published on the subject of normal histology.

---

Lea's Series of Medical Epitomes. Nervous and Mental Diseases. A Manual for Students and Practitioners. With an Appendix on Insomnia. By Joseph Darwin Nagel, M.D., Consulting Physician to the French Hospital, New York. Series edited by V. C. Pedersen, M.D., Instructor in Surgery, New York Polyclinic Medical School and Hospital. Duodecimo, pp. 276, with 46 engravings. Philadelphia and New York: Lea Brothers & Company. 1904. (Price, $1.00.)

The author of this epitome of neurology has succeeded in accomplishing a most difficult task—namely, condensing the essentials of a complex topic into the narrow limits of an examination compend, while at the same time preserving the continuity of the subject. The harmonious relations of the several phases of nervous and mental disease are well preserved and the book meets the purposes for which it was prepared.

---

Lea's Series of Medical Epitomes. Surgery. A Manual for Students and Practitioners. By M. D'Arcy Magee, A.M., M.D., Demonstrator of Surgery and Lecturer on Minor Surgery; and Wallace Johnson, Ph.D., M.D., Demonstrator of Pathology and Bacteriology in Georgetown University Medical School, Washington, D. C. Duodecimo, 295 pages, with 129 engravings. Series edited by V. C. Pedersen, M.D., Instructor in Surgery at the New York Polyclinic Medical School and Hospital. New York, Philadelphia: Lea Brothers & Company. 1904. (Price, $1.00.)

By and large this may be regarded as the best quiz compend of surgery that has been issued. It appears to have profited by others that have preceded it, adopting what in them was useful, rejecting such as was irrelevant, and adding to the whole whatever of importance the several authors deemed expedient.

